# Anti-Inflammatory Effects of RTD-1 in a Murine Model of Chronic *Pseudomonas aeruginosa* Lung Infection: Inhibition of NF-κB, Inflammasome Gene Expression, and Pro-IL-1β Biosynthesis

**DOI:** 10.3390/antibiotics10091043

**Published:** 2021-08-26

**Authors:** Mansour A. Dughbaj, Jordanna G. Jayne, A Young J. Park, Timothy J. Bensman, Marquerita Algorri, Andre J. Ouellette, Michael E. Selsted, Paul M. Beringer

**Affiliations:** 1 Department of Clinical Pharmacy, School of Pharmacy, University of Southern California, 1985 Zonal Avenue, Los Angeles, CA 90033, USA; dughbaj@usc.edu (M.A.D.); jayne@usc.edu (J.G.J.); ayoungpa@usc.edu (A.Y.J.P.); algorri@usc.edu (M.A.); 2 Division of Infectious Disease Pharmacology, Office of Clinical Pharmacology, Office of Translational Sciences, Center for Drug Evaluation and Research, US Food and Drug Administration, Silver Spring, MD 20993, USA; bensman09@gmail.com; 3 Department of Pathology and Laboratory Medicine, Keck School of Medicine, University of Southern California, Los Angeles, CA 90033, USA; aouellet@med.usc.edu (A.J.O.); selsted@med.usc.edu (M.E.S.); 4 Norris Comprehensive Cancer Center, University of Southern California, Los Angeles, CA 90033, USA

**Keywords:** cystic fibrosis, inflammasome, inflammation, host defense peptides, Rhesus theta defensin-1

## Abstract

Vicious cycles of chronic airway obstruction, lung infections with *Pseudomonas aeruginosa*, and neutrophil-dominated inflammation contribute to morbidity and mortality in cystic fibrosis (CF) patients. Rhesus theta defensin-1 (RTD-1) is an antimicrobial macrocyclic peptide with immunomodulatory properties. Our objective was to investigate the anti-inflammatory effect of RTD-1 in a murine model of chronic *P. aeruginosa* lung infection. Mice received nebulized RTD-1 daily for 6 days. Bacterial burden, leukocyte counts, and cytokine concentrations were evaluated. Microarray analysis was performed on bronchoalveolar lavage fluid (BALF) cells and lung tissue homogenates. In vitro effects of RTD-1 in THP-1 cells were assessed using quantitative reverse transcription PCR, enzyme-linked immunosorbent assays, immunoblots, confocal microscopy, enzymatic activity assays, and NF-κB-reporter assays. RTD-1 significantly reduced lung white blood cell counts on days 3 (−54.95%; *p* = 0.0003) and 7 (−31.71%; *p* = 0.0097). Microarray analysis of lung tissue homogenates and BALF cells revealed that RTD-1 significantly reduced proinflammatory gene expression, particularly inflammasome-related genes (nod-like receptor protein 3, Mediterranean fever gene, interleukin (IL)-1α, and IL-1β) relative to the control. In vitro studies demonstrated NF–κB activation was reduced two-fold (*p* ≤ 0.0001) by RTD-1 treatment. Immunoblots revealed that RTD-1 treatment inhibited proIL-1β biosynthesis. Additionally, RTD-1 treatment was associated with a reduction in caspase-1 activation (FC = −1.79; *p* = 0.0052). RTD-1 exhibited potent anti-inflammatory activity in chronically infected mice. Importantly, RTD-1 inhibits inflammasome activity, which is possibly a downstream effect of NF-κB modulation. These findings support that this immunomodulatory peptide may be a promising therapeutic for CF-associated lung disease.

## 1. Introduction

Cystic fibrosis (CF) is an autosomal recessive disorder characterized by mutations in the CF transmembrane conductance regulator gene (*CFTR*). Over 1900 mutations can lead to defective mucus secretion, primarily in the respiratory and gastrointestinal systems [1]. *CFTR* dysfunction results in defects in ion and water transport and increased mucus viscosity that then leads to destructive cycles of airway obstruction, inflammation, and infection contributing to bronchiectasis and progressive loss of lung function [2].

Lung disease in CF is characterized by neutrophil-dominated airway inflammation with excessive concentrations of proteases (e.g., neutrophil elastase) and proinflammatory cytokines (i.e., tumor necrosis factor [TNF], interleukin (IL)-1, IL-6, and IL-8), which are associated with lung disease progression and shortened survival [3]. In particular, clinical studies have shown that IL-1β concentrations are significantly increased in the bronchoalveolar lavage (BAL) and sputum of people with CF, and IL-1β polymorphisms are linked with disease severity [4,5,6,7]. Emerging data indicates the inflammasome may play an important role in CF inflammatory disease as the lack of extracellular chloride due to a CFTR dysfunction may increase nod-like receptor protein 3 (NLRP3) stimulation and maturation of IL-1β [8,9]. Clinical studies with high-dose ibuprofen and oral corticosteroids have demonstrated reduced rates of pulmonary function decline; however, their clinical use is limited due to significant adverse effects [10,11,12]. Azithromycin is frequently prescribed to patients with CF, however, its anti-inflammatory effects are modest and may not be sustained [13,14,15]. Therefore, there is a critical need to develop safe and efficacious anti-inflammatory therapies to treat lung disease in CF.

Cationic host defense peptides (HDPs) exhibit both antimicrobial and immunomodulatory properties [16,17]. Several cationic HDPs are effective in murine models of endotoxin-induced lung injury [18,19] and in a *P*. *aeruginosa* alginate chronic infection model [20,21,22]. Rhesus theta defensin-1 (RTD-1) is a macrocyclic HDP endogenously expressed in leukocytes of Old World monkeys. This macrocyclic peptide exhibits broad spectrum in vitro antibacterial activity that includes activity against multidrug-resistant *P. aeruginosa* isolates from people with CF [22,23]. RTD-1 provides a unique immunomodulatory mechanism of action compared with other cationic HDPs in which the anti-inflammatory action is an indirect effect of endotoxin neutralization [24,25]. Previous investigations found that the anti-inflammatory activity of RTD-1 is mediated in part by the inhibition of p38 mitogen-activated protein kinase (MAPK) phosphorylation, NF-κB nuclear translocation via the phosphorylation of protein kinase B (AKT) and inhibition of IκBα degradation [24], and inhibition of TNF converting enzyme (TACE/ADAM17) [26]. In vivo studies have indicated that RTD-1 has potent antimicrobial and immunomodulatory properties in murine models of acute lung injury [19], chronic lung infection [21], sepsis [25], systemic candidiasis [27], and rheumatoid arthritis [28].

In the current investigation, we sought to further examine the anti-inflammatory activity of RTD-1 in the context of chronic *P. aeruginosa* lung infection [29,30]. We assessed in vivo anti-inflammatory effects by evaluating white blood cell counts (WBCs) and cytokine/chemokine secretions in bronchoalveolar lavage fluid (BALF). We also performed microarray analysis on RNA extracted from murine cells obtained from BALF and lung tissue homogenates. In vitro mechanistic studies utilizing human and murine monocytes/macrophages investigated the effects of RTD-1 on cytokine production, inflammasome activation, gene expression, NF-κB modulation, and transcript stability. These studies confirmed published data that RTD-1 exhibits anti-inflammatory activity through NF-κB inhibition and provided new findings that indicate RTD-1 dampens NLRP3 inflammasome activation and associated cytokines, likely through modulation of NF-κB pathways. Overall, these findings support further investigation of this immunomodulatory peptide as a promising potential therapeutic for the treatment of CF-associated lung disease.

## 2. Methods

### 2.1. In Vivo Studies

#### 2.1.1. Chronic Murine Infection Model

Male 10- to 12-week-old C57BL/6 mice (Charles River Laboratories, Wilmington, MA, USA) weighing 23–29 g were housed in a pathogen-free environment at 22–24 °C and 60–65% humidity with a 12 h light:12 h dark photoperiod. The mice received ad libitum access to standard normal chow. Chronic lung infection was established via intratracheal instillation of *P. aeruginosa* (RP73, mucoid isolate from a patient with CF) embedded in agarose beads. After overnight storage at 4 °C, 50 µL *P. aeruginosa* beads containing 5 × 10^5^ colony forming unit in phosphate-buffered saline (PBS) was instilled intratracheally in each mouse. Previous studies have validated the viability of overnight stored *P. aeruginosa* beads (data not shown). After 24 h of infection, each animal received a 1 h daily treatment with aerosolized saline (*n* = 8) or a delivered dose of 167 μg/kg of RTD-1 (*n* = 8) for 3 or 7 days. Previous dose-ranging studies determined that 167 µg/kg exhibited the optimal anti-inflammatory effect [21]. Details of the nose-only inhalation exposure system used, aerosol characterization of the drug, and dose delivery have been previously published [21].

Mice were euthanized on day 3 or 7. The lungs were washed twice with 0.8 mL PBS containing mini protease inhibitors (cOmplete™, Millipore Sigma, Burlington, MA, USA). WBCs isolated from BALF were stained using Turk Blood Diluting Fluid (Ricca Chemical Company, Arlington, TX, USA) and quantified using a hemocytometer. Cell differential counts were performed after staining using Diff-Quick. Cytokine quantifications were determined from the BALF using the Luminex Bead Array (Luminex, Austin, TX, USA).

The total lung bacterial burden was quantified by plating serially diluted BALF and homogenized lung tissue samples onto *Pseudomonas* isolation agar (agar, 13.6 g/L, magnesium chloride, 1.4 g/L, peptic digest of animal tissue, 20 g/L, potassium sulfate, 10 g/L, triclosan (Irgasan), 0.025 g/L) (Millipore Sigma). Total bacterial lung burden was determined from summation of the BALF and lung tissue homogenate colony counts. 

#### 2.1.2. RNA Microarray Analysis

Samples from lung tissue homogenates and BALF cell pellets were stored in an RLT lysis buffer (Qiagen, Hilden, Germany) containing 1% β-mercaptoethanol (Millipore Sigma). RNA was isolated using RNeasy Mini kit on-column extraction with DNase-1 digestion (Qiagen). Gene expression was determined on the Mouse ref 8 v2.0 Expression BeadChip (Illumina, San Diego, CA, USA). Statistical analysis of differentially expressed genes in the different tissue samples was performed using the gene expression workflow in Partek GS. To identify the most significant differentially expressed genes, lists were generated using the criteria of a false discovery rate corrected using the Benjamini–Hochberg step-up procedure (*p* < 0.05) to account for multiple testing and the differences in mean gene transcript levels were required to be at least 1.5-fold in either direction. 

### 2.2. In Vitro Studies

#### 2.2.1. Cell Culture

Epithelial cells (NuLi and CuFi [ATCC, Manassas, VA, USA]) were cultured in Bronchial Epithelial Cell Growth Medium Bulletkit (Lonza, Walkersville, MD, USA) and inflammation was induced by *P. aeruginosa* filtrate. THP-1 (ATCC) monocytes and MHS macrophages (ATCC) were cultured in Roswell Park Memorial Institute (RPMI) 1640 medium, 2 mM L-glutamine, 25 mM HEPES (Corning Inc., New York, NY, USA) + 10% heat-inactivated fetal bovine serum (HI-FBS; Genesee Scientific, San Diego, CA, USA). THP-1 macrophage-like cell differentiation was induced by treatment with 100 nM phorbol-12-myristate-13-acetate (PMA; Millipore Sigma) and cultured for 48 h. Cells were cultured at 37 °C in 5% CO_2_. Cell lines were found to be mycoplasma-negative using MycoAlert (Lonza). Experiments were performed using THP-1 cells cultured between 8 and 18 passages. RTD-1 was solubilized at a concentration of 10 mg/mL in 0.01% acetic acid. At the initiation of each experiment, the cells were cultured in media containing 1% FBS. Inflammation in THP-1 cells was induced using 100 ng/mL *P. aeruginosa* lipopolysaccharide (LPS; InvivoGen, San Diego, CA, USA). Cells were then immediately dosed with RTD-1 (7.14 μg/mL), azithromycin (50 μg/mL; Millipore Sigma) or MCC950, an NLRP3 inhibitor, (10 μM; Cayman Chemical Company, Ann Arbor, MI, USA) at the beginning of the experiment. Both RTD-1 and azithromycin concentrations were determined based on physiological concentrations observed in the lung epithelial lining fluid in vivo [22,31]. For RNA collection, cells were seeded at 1 × 10^6^ cells per well in 24-well plates and harvested after 3 h. For secreted protein and NF-κB reporter analysis, cells were seeded at 4 × 10^5^ per well in 24-well plates and harvested after 24 h. For Western blot analysis, cells were seeded at 1 × 10^6^ per well in 12-well plates and lysed immediately after the 24 h treatment. To induce inflammasome activation, THP-1 cells were exposed to 5 mM ATP for the final 30 min of the experiment. To perform the NLRP3 destabilization assays, THP-1 macrophages were stimulated and treated with RTD-1 (7.14 μg/mL) for 3 h. A set of cells was exposed to actinomycin-D (5 μg/mL) for 45 min before the end of the 3 h treatment. All experiments were conducted in triplicate on three different days. 

#### 2.2.2. NF-κB Reporter Assay

The THP1-Lucia™ NF-κB reporter cell line (InvivoGen) was used to assess the effect of RTD-1 on NF-κB activation. The cells were starved for 4 h in RPMI 25 mM HEPES 1% FBS before a 16 h overnight treatment period. NF-κB-induced luciferase expression in the cell culture supernatant was quantified using the luciferase detection reagent QUANTI-Luc™ (InvivoGen). Luminosity was measured using a Synergy H1 kinetic fluorescent microplate reader (Bio-Tek, Winooski, VT, USA).

#### 2.2.3. Gene Expression Analysis

Cells (THP-1 monocytes/macrophages, MHS macrophages) were lysed in PureLink (Thermo Fisher, Waltham, MA, USA) spin column-based kit lysis buffer containing 1% β-mercaptoethanol and total RNA isolated according to the PureLink manufacturer’s protocol. cDNA was transcribed using iScript™ Reverse Transcription Supermix for quantitative reverse transcription PCR (RT-PCR; Bio-Rad Laboratories, Hercules, CA, USA). Quantitative realtime PCR assays were performed using SsoAdvanced™ Universal SYBR^®^ Green Supermix (BioRad, Hercules, CA, USA) per the manufacturer’s instructions on the CFX96™ Real-Time PCR Detection System (BioRad). Inflammatory cytokines associated with CF-associated lung disease and inflammasome-related genes were assessed. The primers (Millipore Sigma) used are presented in Supplementary Materials Table S1 [3]. Transcript copy numbers were normalized to the housekeeping genes, *GAPDH* and *RPL27*, using the ΔΔCt method. Gene expression was calculated based on relative fold change. Inflammasome-related gene expression profiles were analyzed with THP-1 macrophages using RT-Profiler Human Inflammasome plates (Qiagen). The data were analyzed using the ΔΔCt method included in the Qiagen Data Analysis Center.

#### 2.2.4. Enzyme-Linked Immunosorbent Assay (ELISA)

Cell culture supernatants were collected at 24 h and stored at −80 °C. Cytokines IL-1β, TNF, IL-6, and CXCL10 were quantified using the V-Plex ELISA (Meso Scale Diagnostics, Rockville, MD, USA) according to the manufacturer’s protocol.

#### 2.2.5. Immunoblot Analyses

THP-1 monocytes were harvested, and the cell pellet was washed twice with ice cold PBS and lysed with Mammalian Protein Extraction Regent lysis buffer (Millipore Sigma) containing c0mplete™ mini protease inhibitors. Protein contents of cellular lysates were quantified using a BCA protein assay kit (Thermo Fisher). Standardized total protein (10 μg) from each treatment was separated on a 4–15% precast polyacrylamide gel (BioRad). Immunoblots were probed using (1:1000) rabbit monoclonal antibodies that target human IL-1β, caspase-1, and GAPDH proteins (Cell Signaling, Danvers, MA, USA). The immunoblot was developed using anti-rabbit HRP-conjugated secondary antibodies and detected by chemiluminescence. Images were acquired using a Chemidoc MP Imager (BioRad). Western blots were normalized to GAPDH and divided by the comparator (stimulated cells). Densitometry analyses were performed using ImageJ software.

#### 2.2.6. Caspase-1 Activity

THP-1 cells were seeded into 96-well plates at a concentration of 40,000 cells per 100 μL complete media. To visualize the role of RTD on inflammasome activation, cells were seeded onto 12 mm glass coverslips (Neuvitro Corporation, Vancouver, WA, USA) in a 24-well plate at a concentration of 2 × 10^5^ cells in 0.5 mL complete medium for confocal imaging. Cells were differentiated in the wells for 48 h in 100 nM PMA. The complete medium was aspirated and replaced with RPMI +1% HI-FBS before addition of LPS (100 ng/mL), RTD-1 (7.14 μg/mL), or azithromycin (50 μg/mL) for 6 h. ATP (5 mM; Millipore Sigma) was added 45 min before cells were labeled using the FAM-FLICA^®^ Caspase-1 Assay Kit (ImmunoChemistry Technologies, Bloomington, MN, USA) according to the manufacturer’s instructions. Inflammasome activity was measured using a Synergy H1 kinetic fluorescent microplate reader. Cells seeded onto the coverslips were fixed and mounted using a Fluoroshield Mounting Medium (Abcam, Cambridge, UK). Representative confocal images were taken using a ZEISS LSM 880 with Airyscan (ZEISS, Oberkochen, Germany). The images were analyzed using ZEN Blue Microscope software (ZEISS). Lastly, RTD-1′s direct effect on caspase-1 inhibition was assessed in vitro using a fluorogenic substrate assay according to the manufacturer’s instructions (Abcam) using a Synergy H1 kinetic fluorescent microplate reader.

#### 2.2.7. Data and Statistical Analysis

Statistical and graphical analysis were performed using GraphPad Prism version 8.0.1 software. A statistical significance level of *p* < 0.05 was determined before analysis. 

## 3. Results

### 3.1. In Vivo Efficacy of Aerosolized RTD-1 in Mice with Chronic Pseudomonas aeruginosa Lung Infection

**Figure 1:**
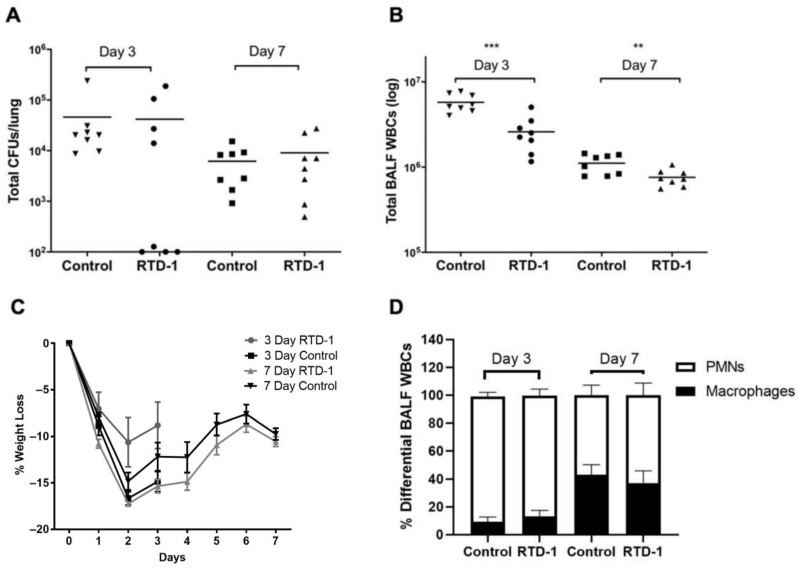
Pathophysiological Studies of Chronic Pseudomonas aeruginosa Lung Infection in Mice Treated with Aerosolized RTD-1. Chronic infection was established via intratracheal instillation of 5 × 10^5^ CFUs of P. aeruginosa (RP73, mucoid CF isolate). Aerosolized RTD-1 at a dose of 167 mg/kg commenced 24 h after infection and continued for 6 days. Effects of treatment on: (**A**) lung bacterial burden, (**B**) total immune cell counts, (**C**) weight, and (**D**) differential cell counts were evaluated (** *p* ≤ 0.01, *** *p* ≤ 0.001). BALF, bronchoalveolar lavage fluid; CFU, colony forming unit; PMN, polymorphonuclear leukocyte; WBC, white blood cell.

We initiated an in vivo study to characterize the efficacy of aerosolized RTD-1 by examining its effects on: (1) immune cell lung infiltration, (2) BALF cytokines/chemokines, and (3) total lung bacterial burden. The results for the differences in antibacterial and anti-inflammatory effects between RTD-1-treated and control mice are presented in Figure 1. No differences in antibacterial effects were found for treatment versus control groups on days 3 or 7 (Figure 1A). Despite the lack of antibacterial effects, RTD-1 treatment was associated with a significant reduction in total WBC counts in the BALF on days 3 (−54.95%; *p* = 0.0003) and 7 (−31.71%; *p* = 0.0097) (Figure 1B). Compared with the control group, mice treated with RTD-1 had reduced weight loss by day 3, but there were no differences on day 7 (Figure 1C). WBC differential counts indicated that neutrophils were predominant on day 3 while an increase in the proportion of macrophages was evident by day 7. However, there were no significant group differences (Figure 1D).

**Figure 2:**
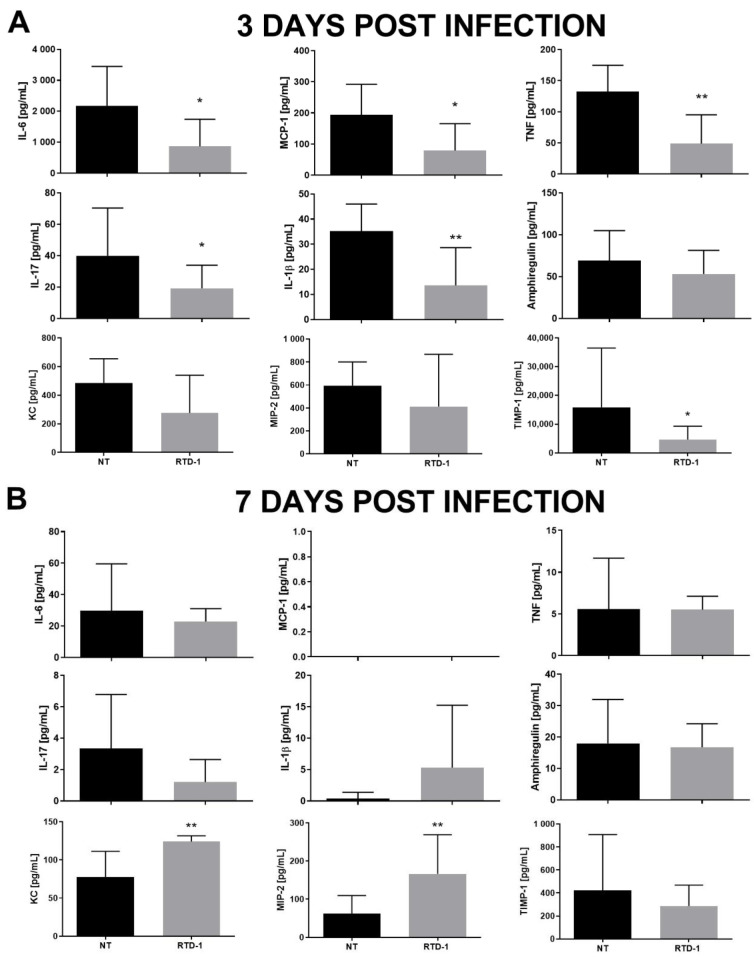
Effect of Aerosolized RTD-1 on BALF Cytokines in Murine Model of Chronic Pseudomonas aeruginosa Lung Infection. Cytokines were quantified from the BALF of C57BL/6 mice chronically infected with P. aeruginosa (RP73). The mice were treated with aerosolized saline or RTD-1 (167mg/kg) for 1 h for 6 days, starting 1 day after bacterial instillation. Cytokines were evaluated on day 3 (**A**) and on day 7 (**B**) (* *p* ≤ 0.05, ** *p* ≤ 0.01). BALF, bronchoalveolar lavage fluid; NT, untreated.

Consistent with the anti-inflammatory effects of RTD-1, peptide treatment was associated with significant reductions in BALF IL-6, MCP-1, TNF, IL-17, IL-1β, and TIMP-1 on day 3 (Figure 2A). On day 7, there were no significant differences between the treatment and control groups for most of the inflammatory biomarkers, apart from increases in KC/CXCL1 and MIP-2/CXCL2 in the RTD-1 treatment group (Figure 2B). Cytokine levels declined in both treated and control mice between days 3 and 7 which is consistent with previously published data demonstrating a decline in lung bacterial burden after day 3 before stabilizing at day 7 [30].

### 3.2. Biological Targets of Anti-Inflammatory Activity of RTD-1 during Chronic Pseudomonas aeruginosa Lung Infection

To investigate the anti-inflammatory mechanism of RTD-1 in vivo, we performed a microarray analysis using RNA isolated from lung tissue homogenates and the BALF cell pellets of RTD-1-treated and control mice that were treated for 3 days. Using Ingenuity Pathway Analysis (IPA), we identified networks affected by RTD-1 in lung tissue homogenates and BALF cells. IPA identified the most relevant biological functions affected by RTD-1 activity. These IPA analyses indicated that RTD-1 reduced cell-to-cell signaling and interaction, hematological system development and function, and the inflammatory response. In lung tissue, the highest scoring networks clustered around TNF. Differential gene expression analysis showed that RTD-1 treatment significantly altered the expression (2-fold up or down; unadjusted *p*-value < 0.05) of 109 genes in BALF immune cells and 58 genes in lung tissue homogenates. The most affected were inflammation-associated genes, which were largely downregulated in the RTD-1-treated group. 

Global gene expression analysis of samples from the saline control and RTD-1-treated mice were compared using volcano plots (Supplementary Materials Figure S1). Although most affected genes were downregulated, some genes in the lung tissue homogenates and BAL cells were upregulated (>2-fold change [FC]). The results indicated that RTD-1 upregulated genes encoding cytochrome P450 enzymes, including *Cyp2F2* and *Cyp2A5* in BALF cells, by 2.555-fold and 2.148-fold, respectively; *Cyp2E1* was upregulated 2.019-fold in lung tissue homogenates. RTD-1 also induced a 2.307-fold upregulation of the organic anion uptake transporter, *SLCO2B1* (*OATP2B1*) in BALF cell pellets. In lung tissue homogenates, RTD-1 increased expression of *BC055107* (*FAM107a*, *DDR1*) by 2.497-fold. This stress-inducible actin-binding protein facilitates regulation of filamentous actin dynamics [32]. We also found a 2.562-fold increase in *NPR3*. This gene encodes a natriuretic peptide receptor that helps to regulate blood pressure, blood volume, and pulmonary hypertension [33].

The top 20 genes affected by RTD-1 treatment in BALF immune cells and lung tissue homogenates are presented in (Figure S2A). For the lung tissue homogenates, 17 genes were downregulated, and three genes were upregulated (Figure S2B). We found 26 genes in common between the two tissues that were significantly altered by RTD-1 treatment; 25 genes were significantly downregulated (Figure S2C). RTD-1 induced large fold change reductions in the proinflammatory cytokines *CXCL10* (lung −4.4; BALF −3.45), *CXCL9* (lung −2.78; BALF −2.33), *CXCL2* (lung −2.81; BALF −3.22), *IL-1β* (lung −2.61; BALF −3.27), and *IFNγ* (lung −2.45; BALF −2.00) compared to the control treatment group. *FKBP5* was the only gene significantly upregulated in both tissues (BALF 2.68; lung −2.44). FKBP5 is an important stress response modulator and a cochaperone of heat shock protein-90 (HSP90) [34,35].

Overall, inflammation-associated genes were significantly reduced, including genes overexpressed in airways of individuals with CF (e.g., *TNF* and *IL-1β*). In particular, these results identified several inflammasome-related genes that were modulated by RTD-1 treatment in both tissues (Figure S3). Inflammasome machinery and cytokines including *NLRP3* (lung −1.2; BALF −2.21), Mediterranean fever gene (*MEFV*) (lung −2.10; BALF −2.27), *IL-1α* (lung −2.6; BALF −3.03), and *IL-1β* (lung −2.61; BALF −3.27) were significantly downregulated by RTD-1 treatment compared to the control.

### 3.3. RTD-1 Inhibits NF-κB Activity 

**Figure 3:**
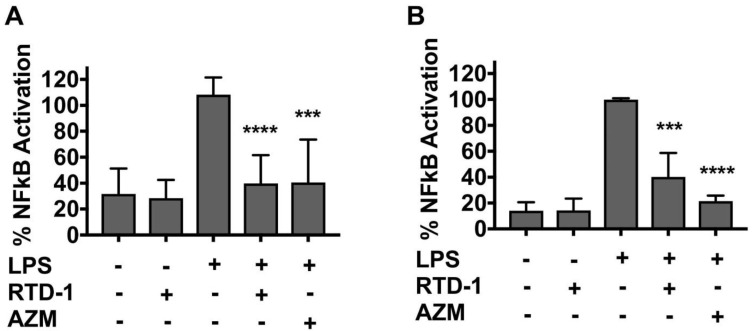
Inhibition of NF-κB by RTD-1. THP-1 Lucia NF-κB reporter monocytes (**A**) and macrophages (**B**) were treated with LPS (100 ng/mL) and RTD-1 (7.14 µg/mL) or azithromycin (50 μg/mL) for 16 h. The x-axis denotes the presence (+) or absence (−) of each compound. Luminescence signal intensity was measured using the Lucia NF-κB reporter gene assay and calculated as the percentage of treated cells over control cells. Results were normalized to LPS-stimulated cells (mean ± standard deviation values). Statistical significance between LPS-challenged and RTD-1 and azithromycin treatments was evaluated using two sample t-tests (**** *p* ≤ 0.0001, *** *p* ≤ 0.001). AZM, azithromycin; LPS, lipopolysaccharide.

Due to its importance as a central regulator of inflammatory genes, we evaluated the effect of RTD-1 treatment on NF-κB activity of THP-1 monocytes and macrophages. Macrophages are also key contributors to CF-associated lung pathology and epithelial dysfunction via elevated secretion of proinflammatory cytokines and elastase [36]. A two-fold inhibition of NF-κB activity occurred after RTD-1 treatment in LPS-stimulated THP-1 monocytes and macrophages (Figure 3A,B), which was consistent with previously published data [24]. Azithromycin appeared to inhibit NF-κB to a greater extent than RTD-1 in the THP-1 macrophages (Figure 3B); however, the difference was not statistically significant, and was not observed in THP-1 monocytes (Figure 3A).

### 3.4. RTD-1 Inhibits Inflammatory Gene Expression 

To further evaluate the anti-inflammatory effects of RTD-1, we conducted in vitro experiments in LPS-stimulated THP-1 monocytes and bronchial epithelial cells. Study results suggested that hematopoietic cells are the predominant source of inflammasome-induced IL-1β secretions within lungs affected by CF [8]; however, pilot experiments using submerged NuLi and CuFi cell lines revealed limited LPS-induced expression of inflammatory genes (and proteins). Previously, we observed that RTD-1 downregulated several inflammatory cytokines in murine MHS macrophages, including macrophage inflammatory protein (MIP)-2, monocyte chemoattractant protein (MCP)-1, IL-6, and TNF [19]. Thus, we focused the research on human THP-1 cells. In comparison to untreated cells, RTD-1 significantly increased the transcription of *IL-8*, *IL-6* and *CXCL10* (Figure S5D–F). In contrast, RTD-1 significantly inhibited *IL-1β*, *TNF*, *IL-1α*, *IL-8*, and *IL-6* (Figure S5A–E) in LPS-stimulated cells when compared to untreated LPS-stimulated cells. RTD-1 did not affect *CXCL10* expression in LPS-stimulated cells (Figure S5F). This finding contrasts with the in vivo data in this report (Figure S2) as well as previous findings that demonstrate RTD-1 potently inhibited *CXCL10* mRNA in murine alveolar macrophages (Figure S4A). This reason for this apparent discrepancy is unknown but appears to be unique to the immortalized THP-1 cells.

RTD-1 exhibited activity similar to azithromycin across the cytokine panel with the exception of CXCL10, where azithromycin treatment resulted in a significant inhibition of *CXCL10* mRNA (3.35-fold reduction) compared to treatment with RTD-1 and LPS-stimulated cells (Figure S5F). Cotreatment with RTD-1 and azithromycin resulted in an additional reduction in *IL-8* and *IL-6* mRNA, but not *IL-18* (Figure S5D,E). The results suggested that RTD-1 and azithromycin exert anti-inflammatory activities through alternative, but complementary mechanisms. 

### 3.5. RTD-1 Treatment Reduces Cytokine Production In Vitro

To determine if the downregulation of inflammatory genes was accompanied by a decrease in corresponding protein levels, we assessed cytokine production in LPS-stimulated THP-1 monocytes with and without peptide and drug treatment. RTD-1 treatment reduced IL-1β protein secretion by approximately 10-fold, which was comparable to the effect of RTD-1 on *IL-1β* gene mRNA levels (Figure S6A). IL-1α protein concentrations were below the lower limit of detection for these experiments. RTD-1 treatment had similar inhibitory activity when compared to azithromycin and the NLRP3 inhibitor MCC950 (Figure S6A). RTD-1 potently inhibited TNF secretion with a 5.44-fold reduction (*p* = 0.0002). When coadministered with azithromycin, there was a trend towards greater TNF inhibition relative to RTD-1 or azithromycin alone (Figure S6B) suggesting that RTD-1 and azithromycin inhibit TNF protein secretion through different but complementary mechanisms of action. This mechanistic difference is likely attributable to the pleiotropic inhibitory activities of RTD-1 against TACE/ADAM17, p38 MAPK phosphorylation and NF-κB [26]. Although the combination of RTD-1 and azithromycin resulted in additional inhibition of *IL-6* mRNA compared to each treatment alone, the inhibition did not translate at the protein level (Figure S6C). Consistent with the effect on mRNA levels, RTD-1 did not reduce CXCL10 protein secretion below that of LPS alone (Figure S6D). The difference in CXCL10 secretion is unique to THP-1 cells as MHS murine alveolar macrophages and CuFi bronchial epithelial cells treated with RTD-1 were found to significantly reduce CXCL10 secretion after LPS or *P. aeruginosa* filtrate stimulation, respectively (Figure S4B,C) which is consistent with the in vivo data.

### 3.6. RTD-1 Downregulates Inflammasome-Associated Gene Expression

**Figure 4:**
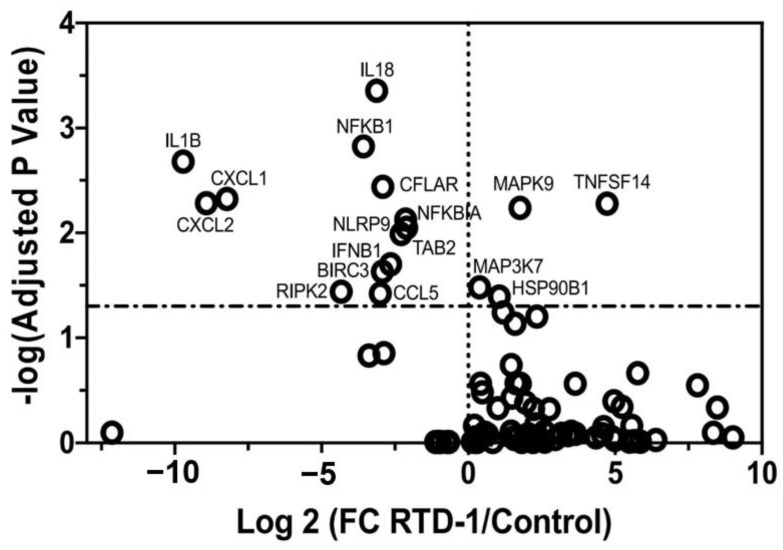
Inhibition of Inflammasome-associated Gene Expression by RTD-1 Treatment. RTD-1 (7.14 μg/mL) inhibited inflammasome-associated genes in LPS (100 ng/mL)-stimulated THP-1 macrophages. RTD-1 reduced expression of downstream inflammasome molecules. The mRNA transcription profiles obtained using qRT-PCR profiler array were plotted according to the log2-fold change and a log-adjusted p-value. Treatment differences were analyzed using the multiple t-test function in Prism 8.0 software. Statistically significant gene expression changes are represented by data above the dotted horizontal line (*p* < 0.05). FC, fold change; LPS, lipopolysaccharide; qRT-PCR, quantitative reverse transcription PCR.

Microarray expression analysis from the chronic *P. aeruginosa* model indicated that RTD-1 significantly downregulated expression of NLRP3 inflammasome-related genes. Therefore, we examined the effect of RTD-1 on the inflammasome in LPS-stimulated THP-1 monocytes and macrophages using a PCR array. Preliminary experiments in THP-1 monocytes demonstrated minimal *NLRP3* gene expression, compared with THP-1 differentiated macrophages. Thus, we assessed the differences in mRNA levels of inflammasome-associated genes in LPS-stimulated THP-1 macrophages. RTD-1 treatment resulted in significantly decreased fold changes of downstream inflammasome-related genes relative to the control treatment group, including *IL-1β* (−8.72), *IL-18* (−2.125), *CXCL1* (−7.225), *CXCL2* (−7.925), *CCL5* (−2.000), and *IFNβ1* (−1.665) (Figure 4). RTD-1 upregulated mitogen-activated protein kinase 9 (*MAPK9*; 2.755) and TNF superfamily member 14 (*TNFSF14*; 5.725) genes.

The microarray analysis revealed that RTD-1 treatment was associated with a significant reduction in *NLRP3* mRNA transcripts. A previous study observed that azithromycin treatment increased the decay of the *NLRP3* transcript over time in THP-1 monocytes [37]. To test the hypothesis that RTD-1 destabilizes the *NLRP3* transcript, the experiment was repeated with LPS-stimulated THP-1 macrophages treated with RTD-1, with or without a 45 min actinomycin-D (a transcriptional inhibitor) treatment. RTD-1 reduced *NLRP3* mRNA levels (−2.17 FC; *p* < 0.0001); however, gene expression between treatment groups with or without actinomycin-D was unchanged, indicating that reduced *NLRP3* mRNA levels was not due to transcript destabilization (Figure S7). 

### 3.7. RTD-1 Decreases IL-1β Precursor Formation

**Figure 5:**
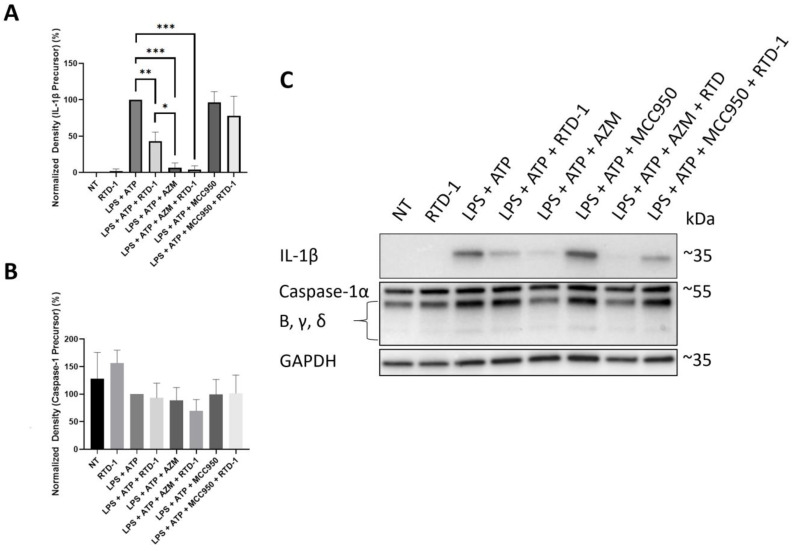
Inhibition of IL-1β Protein Expression. THP-1 monocytes stimulated with 100 ng/mL LPS were simultaneously treated with RTD-1 (7.14 μg/mL) for 24 h and lysed immediately afterwards to determine the effect on expression of (**A**) IL-1β and (**B**) caspase-1 precursor formation. Representative Western blot image is shown in (**C**). Treatment differences were analyzed using t-tests for IL-1β and caspase-1 (* *p* ≤ 0.05, ** *p* ≤ 0.01, *** *p* < 0.0001). AZM, azithromycin; LPS, lipopolysaccharide; NT, No treatment.

To determine if the downregulation of inflammasome-associated genes was accompanied by a decrease in protein expression, we performed immunoblot experiments with RTD-1 in LPS-stimulated THP-1 monocytes and macrophages. Preliminary experiments in THP-1 macrophages demonstrated minimal IL-1β precursor expression, compared with THP-1 monocytes; thus, protein expression was assessed in THP-1 monocytes. RTD-1 and azithromycin significantly inhibited IL-1β precursor (56.98%, *p*-value = 0.0015, and 93.24%, *p*-value ≤ 0.0001, decrease, respectively), compared with cells treated with LPS and ATP alone (Figure 5). The increased activity of azithromycin relative to RTD-1 against IL-1β precursor is likely attributable to its effect on the destabilization of inflammatory mRNA transcripts [37]. Cotreatment with RTD-1 and azithromycin resulted in similar levels of inhibition of IL-1β precursor compared to azithromycin alone. Treatment with RTD-1 or azithromycin, or both, did not result in significant inhibition of caspase-1 protein expression or any other intermediates (Figure 5) indicating that the inhibition of IL-1β precursor by RTD-1 is not mediated by inhibition of caspase-1 activation.

### 3.8. RTD-1 Inhibits Inflammasome Activity

**Figure 6:**
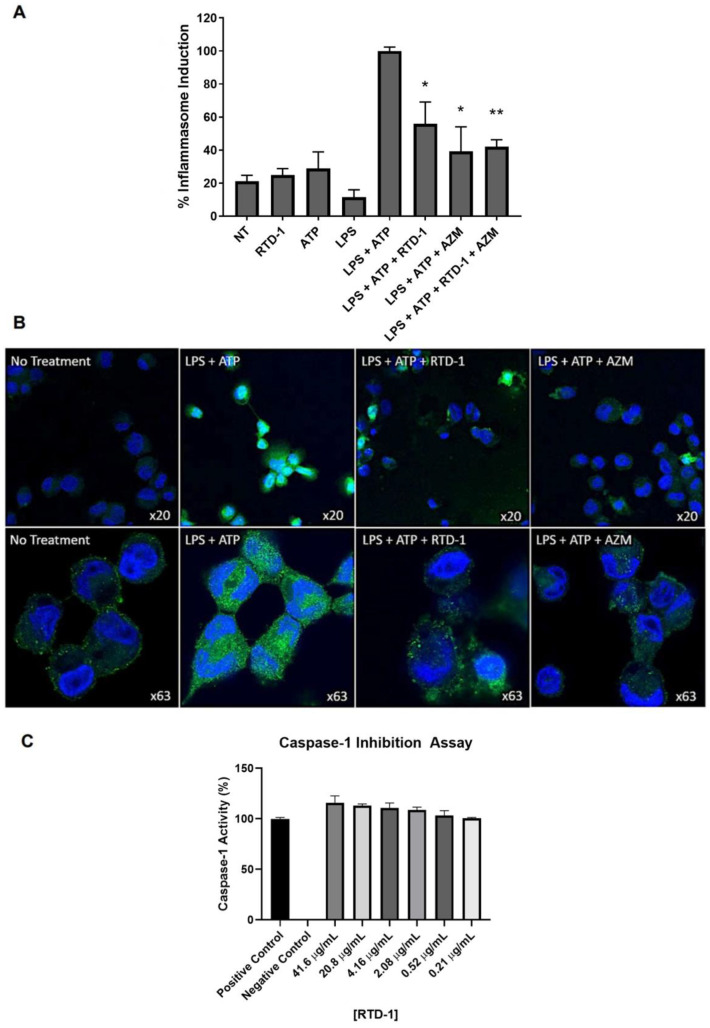
Inflammasome Inhibition by RTD-1 Treatment. (**A**) Caspase-1 activity was determined in THP-1 macrophages stimulated with LPS (100 ng/mL) and RTD-1 (7.14 μg/mL) or azithromycin (50 μg/mL) for 6 h; ATP (5 mM) was added for an additional 45 min. Cells were labelled with FLICA caspase-1 specific monoclonal antibodies and fluorescence was measured. (**B**) Representative confocal images of inflammasome activation are shown. (**C**) The direct effect of RTD-1 on caspase-1 activity using an enzymatic inhibition assay (* *p* ≤ 0.01, ** *p* ≤ 0.001). FLICA caspase-1 specific monoclonal antibody (green), Hoechst 33,342 nuclear stain (blue). ATP, adenosine triphosphate; AZM, azithromycin; LPS, lipopolysaccharide.

To determine if the downregulation of inflammasome-associated genes was associated with functional changes in the inflammasome, we performed inflammasome activation assays (Figure 6A). RTD-1, azithromycin, or their combination resulted in significant fold reductions in caspase-1 activation of −1.79 (*p* = 0.0052), −2.55 (*p* = 0.0022), and −2.38 (*p* = 0.0003), respectively (Figure 6A). Combined treatment with RTD-1 and azithromycin did not result in any additional reduction in inflammasome activity compared to each treatment alone. Representative images of these results are presented in Figure 6B. To further elucidate whether RTD-1 inhibits inflammasome activity directly, we utilized an in vitro caspase-1 inhibitor assay (Figure 6C). We observed no direct caspase-1 inhibition at the highest concentration of 41.6 µg/mL of RTD-1, which suggests the reduction in the inflammasome activity by RTD-1 is possibly a downstream effect of NF-κB modulation.

## 4. Discussion

RTD-1 is an immunomodulatory peptide found to have efficacy in preclinical models of infection and inflammation [19,21,22,25,26,28]. In a murine model of LPS-induced acute lung injury, RTD-1 exhibited a protective effect, as evidenced by the lack of alveolar leukocyte infiltrates, absence of hyaline membranes, and normal alveolar wall thickness [19]. To better characterize the immunomodulatory activity of RTD-1 in the context of chronic *P. aeruginosa* lung infection, we performed RNA microarray analysis of lung tissue homogenates and BALF cells isolated from treated and control mice. Overall, we found that RTD-1 inhibited proinflammatory cytokine gene and protein expression. 

In particular, we report new information demonstrating RTD-1 treatment significantly downregulates the transcription and activation of key inflammasome components. We found that RTD-1 significantly reduced the expression of proinflammatory cytokines (*IL-6*, and *TNF*) and four inflammasome-related genes (*NLRP3*, *MEFV*, *IL-1α*, and *IL-1β*). While most genes were downregulated, *FKBP5* was upregulated in both the mouse lung tissue homogenates and the BALF immune cells. FKBP5 is a molecular cochaperone protein for HSP90 and serves as a modulator of glucocorticoid receptor sensitivity along with a host of other cellular responses [34,35]. HSP90 participates in the regulation of protein kinases, ligases, steroid receptors, cell-cycle regulation, and nuclear transcription factors [38]. Despite upregulation of *FKBP5*, which can induce proinflammatory signaling with HSP90, the overall effect of RTD-1 treatment in chronically infected mice was reduced lung inflammation. 

Furthermore, we observed that RTD-1 may have a role in inflammasome regulation. Treatment with RTD-1 resulted in decreased IL-1β precursor and secreted IL-1β. The gene expression results indicated that RTD-1 reduced *NLRP3* and *IL-1β* mRNA expression. Inflammasome and caspase-1 inhibition studies demonstrated that RTD-1 indirectly inhibited inflammasome activity as caspase-1 activation was reduced. Previous literature indicated NF-κB inhibition was central to the downregulation of NLRP3 and IL-1β protein expression, but not caspase-1 expression [39]. These data suggest that RTD-1 inhibits the priming of inflammasome formation and the subsequent activation of IL-1β and caspase-1, possibly through disruption of NF-κB signaling. 

Accumulating evidence indicates targeting the NLRP3 inflammasome may be an effective treatment for lung infection and inflammation in individuals with CF [40]. *P. aeruginosa* infection induces NLRP3 inflammasome assembly in bronchial epithelial cells isolated from patients with CF [41], THP-1 macrophages, human monocyte-derived macrophages [42], and in F508del–CFTR mice [43]. Results using a murine model of pneumonia indicate that IL-1β and IL-18 participate in the suppression of host capabilities to clear *P. aeruginosa* pulmonary infection [44,45]. In vitro studies found that IL-1β antibody neutralization and inhibition of inflammasome components improve macrophage-dependent *P. aeruginosa* killing and reduce the total bacterial burden [40,42,43]; while the addition of recombinant IL-1β treatment inhibits *P. aeruginosa* clearance [42]. Results of in vivo and in vitro studies indicate that IL-1Ra, an inhibitory protein of various IL-1-related immune responses, can mitigate the damaging effects caused by chronic bacterial colonization [46,47,48]. Inhibition of IL-1β via antibody neutralization [43], small molecule NLRP3 inhibitors including MCC950 [40], inhibition of the epithelial sodium channel-NLRP3 axis [49], or by the IL-1Ra agonist Anakinra [46], ameliorates inflammasome-associated inflammation in models of CF via negative regulation of pathogenic NLRP3 activity. The results of these studies suggest that *P. aeruginosa* exploits the NLRP3 inflammasome pathway to evade immune cell killing and that this characteristic can be used as a therapeutic target. Clinical studies have shown that IL-1β concentrations are significantly increased in the BAL and sputum of people with CF and are negatively correlated to lung function [4,5,6,7]. In addition, IL-1β polymorphisms are linked with disease severity in CF patients [45]. Taken together, the results of this study indicate that RTD-1 may have therapeutic potential as an inflammasome-modulating agent for the CF lung disease.

## 5. Conclusions

In summary, RTD-1 reduced lung neutrophil infiltration and inflammatory cytokine production in a murine model of chronic *P. aeruginosa* lung infection. This study also establishes new findings that RTD-1 significantly downregulated several inflammasome-related genes (*NLRP3*, *MEFV*, *IL-1α*, and *IL-1β*) in vivo. In vitro results confirm the dampening of NLRP3 inflammasome activity of RTD-1 and suggest that the effect is possibly due to a downstream effect of NF-κB inhibition. The anti-inflammatory properties of RTD-1 offer a potential unique therapeutic approach to the treatment of CF lung disease. 

## Data Availability

The datasets used and/or analyzed during this study are available from the corresponding author upon reasonable request.

## Supplementary Materials

The following are available online at https://www.mdpi.com/article/10.3390/antibiotics10091043/s1, Figure S1: Microarray analysis of Lung Tissue and BALF Cells of RTD-1-treated and Saline Control Mice with Chronic *Pseudomonas aeruginosa* Lung Infection, Figure S2: Top Up- or Downregulated Genes in Lung Tissue and BALF cells in RTD-1-treated Mice with chronic *P. aeruginosa* lung infection. The top 20 most highly up- or downregulated genes in BALF cells and lung tissue homogenates following RTD-1 treatment, Figure S3: Inflammasome-associated Gene Expression Changes in Lung Tissue and BALF in RTD-1-treated Mice with Chronic Infection. Microarray Analysis identified a list of inflammasome-associated gene expression changes due to RTD-1 treatment. Blue and red squares indicate down- or upregulation of indicated genes, respectively, Figure S4: RTD-1 Treatment Reduces CXCL10 Transcription and Translation in Different Cell Types. MHS murine alveolar macrophages were stimulated with LPS (100 ng/mL) and RTD-1 (7.14 μg/mL) for 3 h and CXCL10 (A) transcription and (B) translation was evaluated through qRT-PCR and ELISA, respectively. (C) CuFi bronchial epithelial cells were stimulated with *P. aeruginosa* filtrate and cytokine secretion was evaluated. LPS, lipopolysaccharide; Pa, *P. aeruginosa*; NT, No treatment, Figure S5: Effect of RTD-1 Treatment on Inflammatory Gene Expression. The effect of RTD-1 (7.14 μg/mL) on THP-1 monocytes stimulated with 100 ng/mL LPS for 3 h was determined through gene expression analysis of key inflammatory cytokines: (A) IL-1β, (B) TNF-α, (C) IL-1α, (D) IL-8, (E) IL-6 and (F) CXCL10. Gene expression was quantified using qRT-PCR (*n* = 3). Treatment differences were analyzed using t-tests after normalization to the housekeeping genes. Mann-Whitney tests were performed for IL-1α and TNF-α (** *p* ≤ 0.01, *** *p* ≤ 0.001, **** *p* < 0.0001). AZM, azithromycin; FC, fold change; LPS, lipopolysaccharide; NT, No treatment, Figure S6: Effect of RTD-1 Treatment on Inflammatory Cytokine Release. The effect of RTD-1 (7.14 μg/mL) on cytokine expression from THP-1 monocytes stimulated with 100 ng/mL LPS for 24 h: (A) IL-1β, (B) TNF-α (C) IL-6 and (D) CXCL10. Cytokines were quantified using ELISA (*n* = 3). Treatment differences were analyzed using parametric t-tests for cytokines IL-1β and TNF-α. For IL-6 and CXCL10, Mann-Whitney tests were performed. (* *p* ≤ 0.05, ** *p* ≤ 0.01, *** *p* ≤ 0.001, **** *p* ≤ 0.0001). ATP, Adenosine triphosphate; AZM, azithromycin; ELISA, enzyme-linked immunosorbent assay; LPS, lipopolysaccharide, Figure S7: RTD-1 Does Not Destabilize NLRP3 mRNA Transcription in THP-1 Macrophages. NLRP3 mRNA transcription was determined using THP-1 macrophages stimulated with LPS (100 ng/mL) and treated with RTD-1 (7.14 μg/mL) for 3 h. A separate set of cells was also exposed to actinomycin-D (5 μg/mL) for an additional 45 min. ATP, Adenosine triphosphate; LPS, lipopolysaccharide, Table S1: Forward and reverse primer sequences used in qRT-PCR experiments.

## Abbreviations

Adenosine triphosphate (ATP), azithromycin (AZM), bronchoalveolar lavage (BAL), lipopolysaccharide (LPS), Mediterranean fever gene (MEFV), nod-like receptor 3 (NLRP3), Rhesus theta defensin-1 (RTD-1), tumor necrosis factor (TNF), white blood cells (WBC).
