# Supplementary material for: Anti-Inflammatory Effects of RTD-1 in a Murine Model of Chronic Pseudomonas aeruginosa Lung Infection: Inhibition of NF-κB, Inflammasome Gene Expression, and Pro-IL-1β Biosynthesis
Source: Antibiotics (Basel). 2021 Aug 26;10(9):1043. doi: 10.3390/antibiotics10091043 (PMC8466744; doi:10.3390/antibiotics10091043)
Supplement: Supplementary file 1 [file antibiotics-10-01043-s001.zip › antibiotics-1335612-supplementary.pdf]

Supplementary Materials

Anti-inflammatory effects of RTD-1 in a murine model of chronic *Pseudomonas aeruginosa* lung infection: inhibition of NF-κB, inflammasome gene expression, and pro-IL-1β biosynthesis

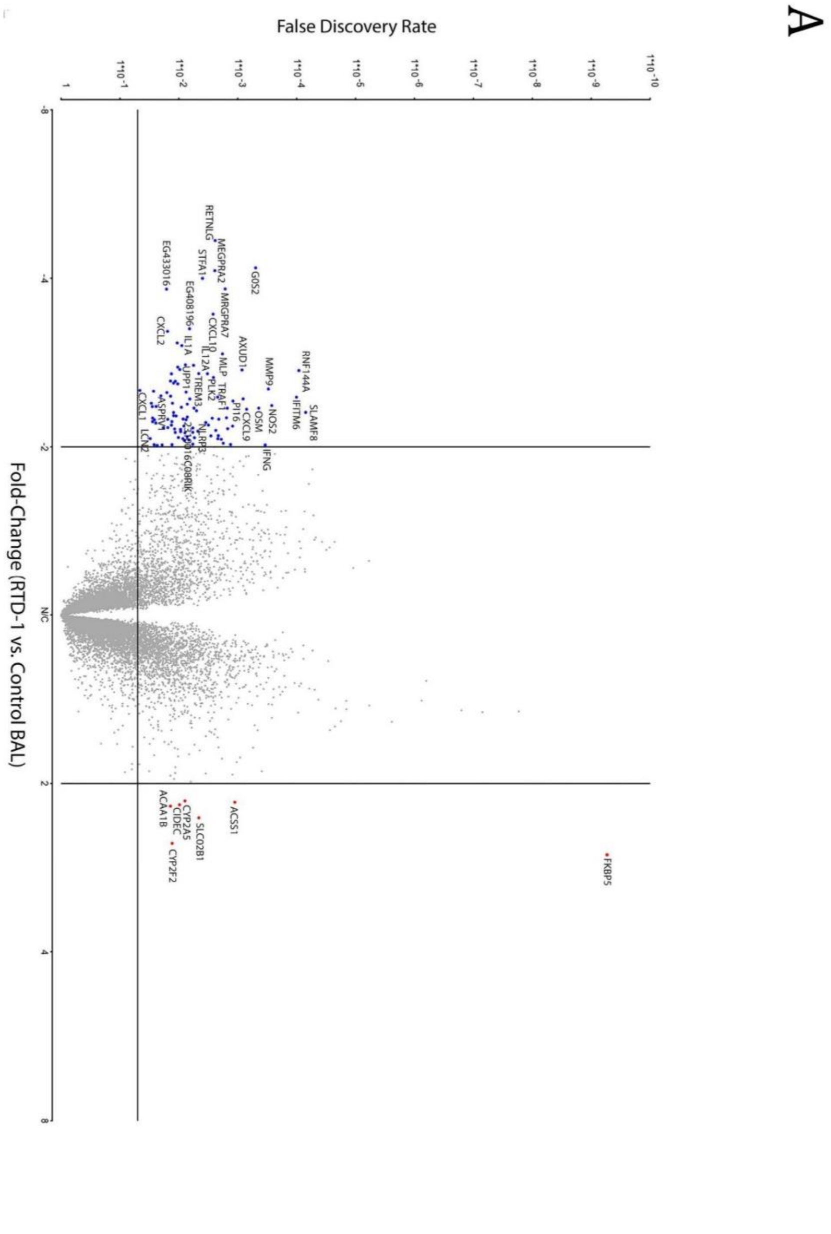

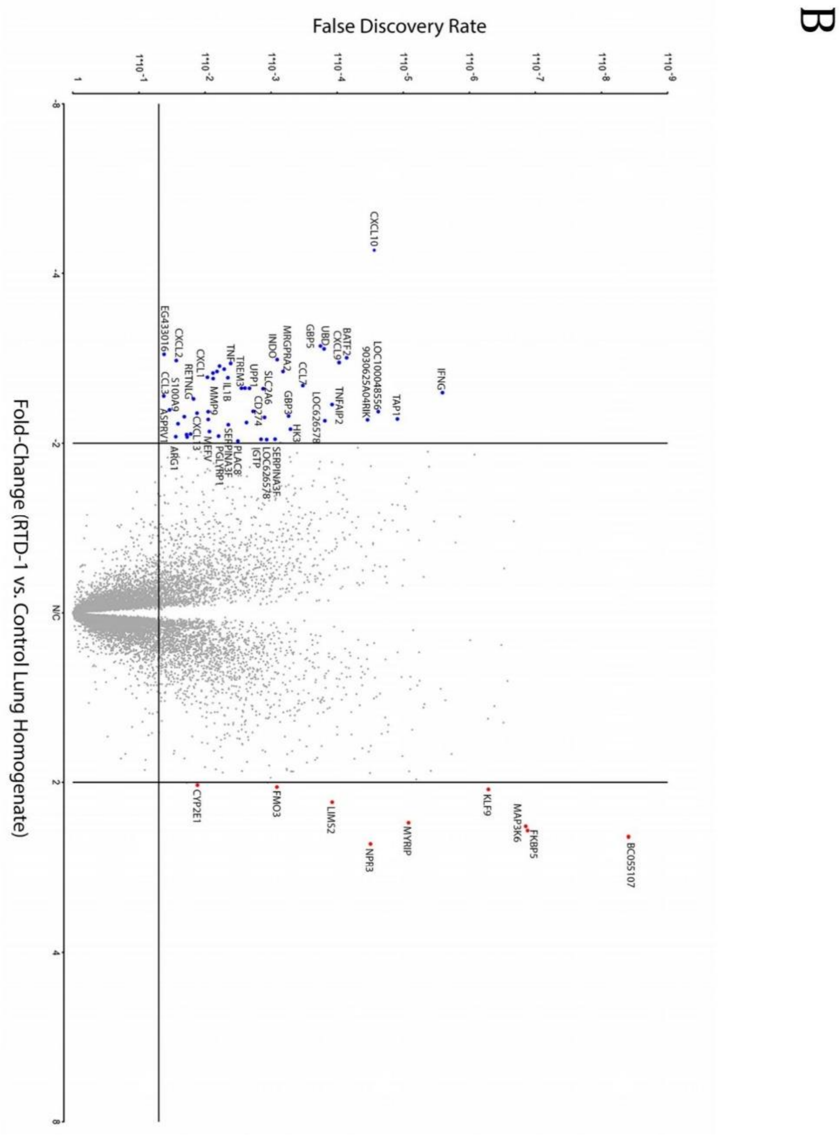

Figure S1. Microarray analysis of Lung Tissue and BALF Cells of RTD-1-treated and Saline Control Mice with Chronic *Pseudomonas aeruginosa* Lung Infection. Volcano plots of microarray data for: **A**) BALF cells and **B**) lung tissue homogenates. The horizontal line indicates the cut-off for an unadjusted *p*-value of 0.05; vertical lines indicate the 2-fold differences in both directions. BALF, bronchoalveolar lavage fluid.

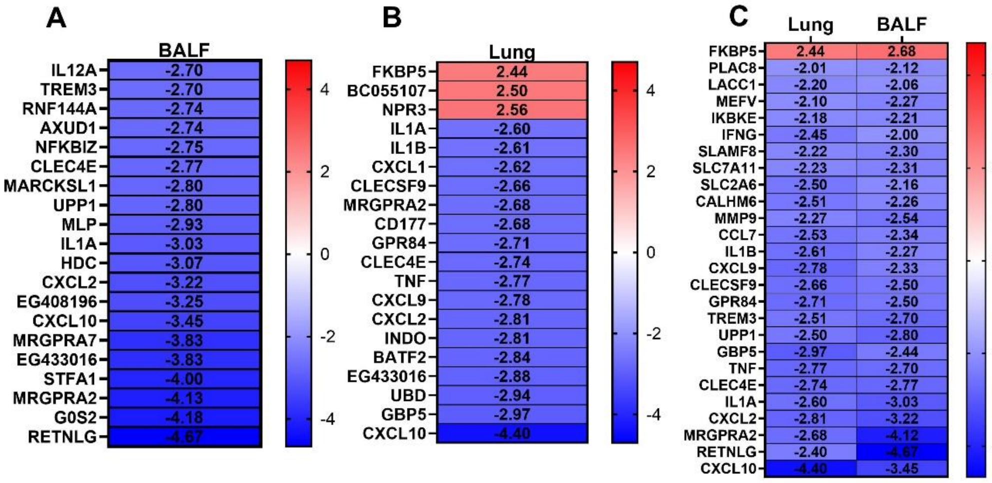

Figure S2. Top Up- or Downregulated Genes in Lung Tissue and BALF cells in RTD-1-treated Mice with chronic *P. aeruginosa* lung infection. The top 20 most highly up- or downregulated genes in BALF cells and lung tissue homogenates

following RTD-1 treatment. Blue and red squares indicate down- or upregulation of indicated genes in: **A**) BALF immune cells or **B**) lung tissue homogenates of mice treated with aerosolized RTD-1, compared with saline-treated controls. **C**) The genes commonly altered by RTD-1 treatment in both lung tissue homogenate and BALF cells. BALF, bronchoalveolar lavage fluid.

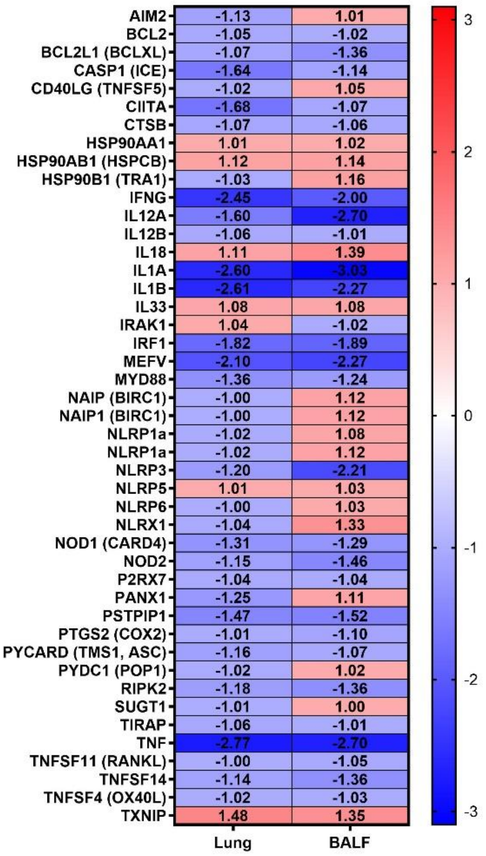

Figure S3. Inflammasome-associated Gene Expression Changes in Lung Tissue and BALF in RTD-1-treated Mice with Chronic Infection**.** Microarray Analysis identified a list of inflammasome-associated gene expression changes due to RTD-1 treatment. Blue and red squares indicate down- or upregulation of indicated genes, respectively.

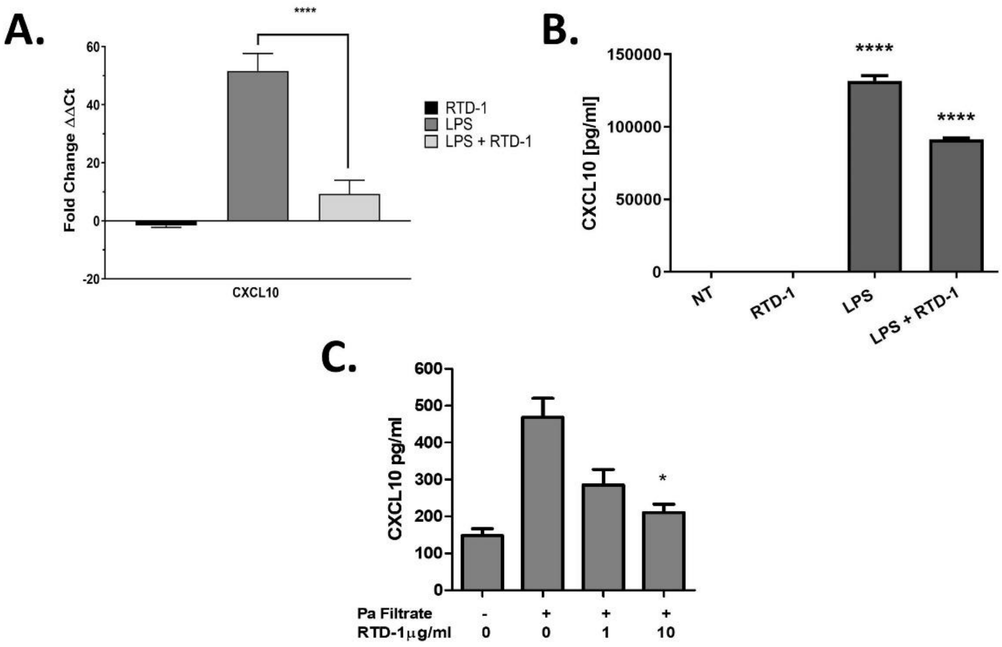

Figure S4. RTD-1 Treatment Reduces CXCL10 Transcription and Translation in Different Cell Types. MHS murine alveolar macrophages were stimulated with LPS (100 ng/ml) and RTD-1 (7.14 μg/ml) for 3 h and CXCL10 **A**) transcription and **B**)

translation was evaluated through qRT-PCR and ELISA, respectively. **C**) CuFi bronchial epithelial cells were stimulated with *P. aeruginosa* filtrate and cytokine secretion was evaluated. LPS, lipopolysaccharide; Pa, *P. aeruginosa*; NT, No treatment.

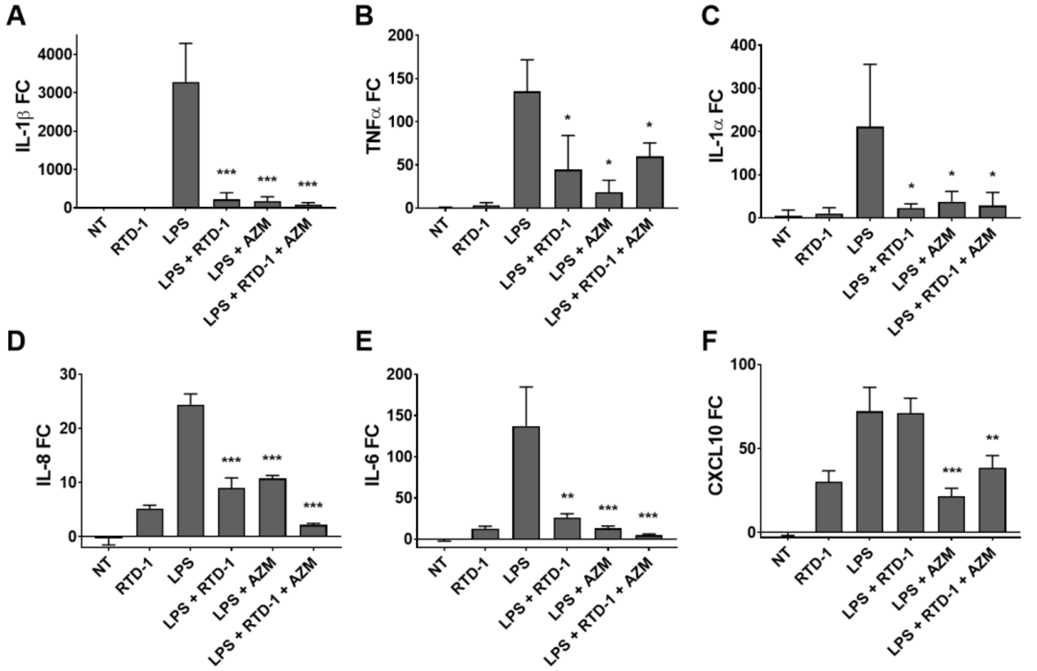

Figure S5. Effect of RTD-1 Treatment on Inflammatory Gene Expression. The effect of RTD-1 (7.14 μg/ml) on THP-1 monocytes stimulated with 100 ng/ml LPS for 3 h was determined through gene expression analysis of key inflammatory cytokines: **A**) IL-1β, **B**) TNF-α, **C**) IL-1α, **D**) IL-8, **E**) IL-6 and **F**) CXCL10. Gene expression was quantified using qRT-PCR (n = 3). Treatment differences were analyzed using t-tests after normalization to the housekeeping genes. Mann-Whitney tests were performed for IL-1α and TNF-α (* $p \leq 0.01$, ** $p \leq 0.001$, *** $p < 0.0001$). AZM, azithromycin; FC, fold change; LPS, lipopolysaccharide; NT, No treatment.

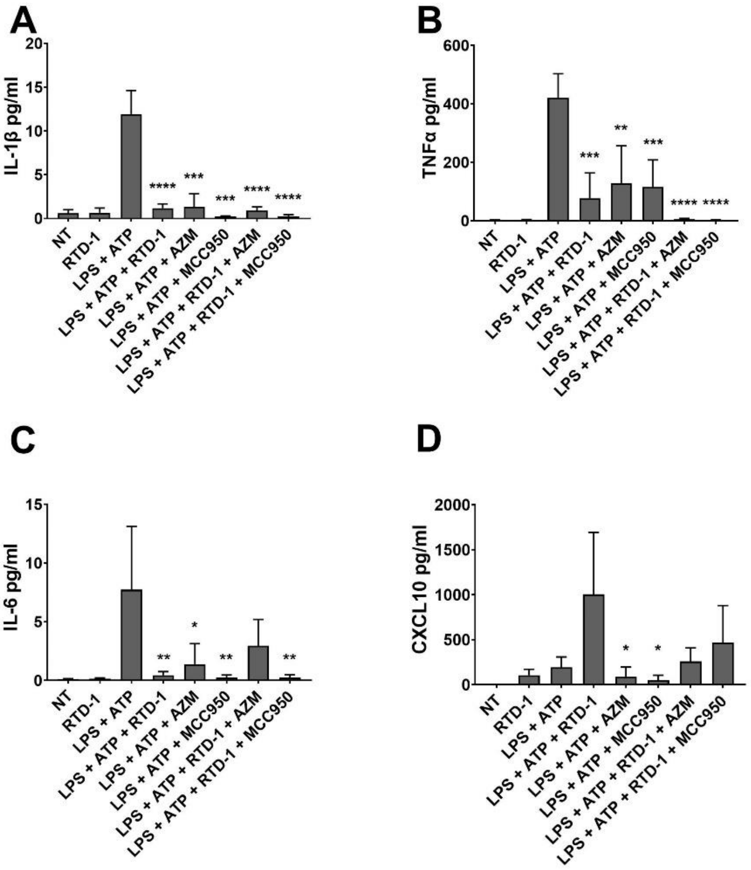

Figure S6. Effect of RTD-1 Treatment on Inflammatory Cytokine Release. The effect of RTD-1 (7.14 μg/ml) on cytokine expression from THP-1 monocytes stimulated with 100 ng/ml LPS for 24 h: **A**) IL-1β, **B**) TNF-*α* **C**) IL-6 and **D**) CXCL10. Cytokines were quantified using ELISA (n = 3). Treatment differences were analyzed using parametric t-tests for cytokines IL-1β and TNF-*α*. For IL-6 and CXCL10, Mann-Whitney tests were performed. (* $p \leq 0.05$, ** $p \leq 0.01$, *** $p \leq 0.001$, ****$p \leq 0.0001$). ATP, Adenosine triphosphate; AZM, azithromycin; ELISA, enzyme-linked immunosorbent assay; LPS, lipopolysaccharide.

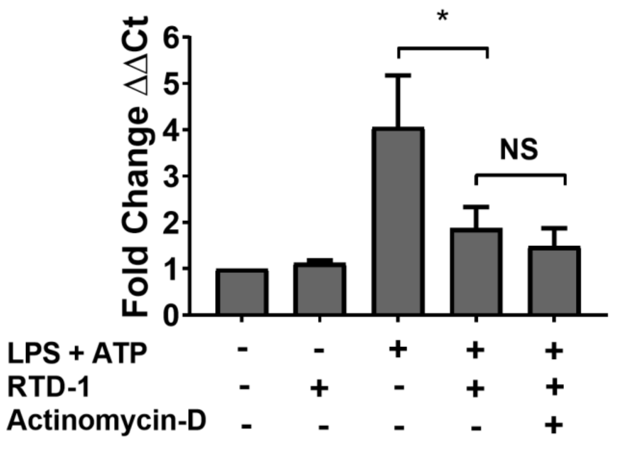

Figure S7. RTD-1 Does Not Destabilize NLRP3 mRNA Transcription in THP-1 Macrophages. The x-axis denotes the presence (+) or absence (–) of each compound. NLRP3 mRNA transcription was determined using THP-1 macrophages stimulated with LPS (100 ng/ml) and treated with RTD-1 (7.14 μg/ml) for 3 h. A separate set of cells was also exposed to actinomycin-D (5 μg/ml) for an additional 45 min. (* $p \leq 0.0001$). ATP, Adenosine triphosphate; LPS, lipopolysaccharide.

Table S1. Forward and reverse primer sequences used in qRT-PCR experiments.

Primer	Forward	Reverse
GAPDH	ACAGTTGCCATGTAGACC	TTGAGCACAGGGTACTTTA
RPL27	CGTCAATAAGGATGTCTTCAG	GTTCTTGCCTGTCTTGTATC
NLRP3	AGGTGTTGGAATTAGACAAC	AATACATTTCAGACAACCCC
IL-1β	CTAAACAGATGAAGTGCTCC	GGTCATTCTCCTGGAAGG
TNF-α	CTATGTCTCAGCCTCTTCTC	CATTTGGGAACTTCTCATCC
IL-8	GTTTTTGAAGAGGGCTGAG	TTTGCTTGAAGTTTCACTGG
IL-6	GCAGAAAAAGGCAAAGAATC	CTACATTTGCCGAAGAGC
CXCL10	CTTCTGAAAGGTGACCAGCC	GTCGCACCTCCACATAGCTT
IL-18	CCTTTAAGGAAATGAATCCTCC	CATCTTATTATCATGTCCTGGG
IL-1α	CATAACCCATGATCTGGAAG	ATTXATGACAAACTTCTGCC
